# Prevalence of *Trichomonas vaginalis* Among Women in the Chinese Population: A Systematic Review and Meta-Analysis

**DOI:** 10.3390/tropicalmed10040113

**Published:** 2025-04-19

**Authors:** Shuang Li, Jiahui Xu, Sisi Ru, Changjun Hu, Chongyang Liu, Xingquan Sun, Heteng Guo, Xi Zhang

**Affiliations:** Department of Parasitology, School of Basic Medical Sciences, Zhengzhou University, Zhengzhou 450001, China; lishuang1619@163.com (S.L.); mars060659509@163.com (J.X.); russ248@163.com (S.R.); cjhu_123456@163.com (C.H.); 19545628954@163.com (C.L.); 13276901836@163.com (X.S.); 18639900659@163.com (H.G.)

**Keywords:** *Trichomonas vaginalis*, trichomoniasis, China, non-viral sexually transmitted infection, systematic review

## Abstract

*Trichomonas vaginalis* (TV) is the most common non-viral sexually transmitted infection (STI) among women worldwide. However, there is little information available regarding the burden of trichomoniasis infection among Chinese women. The aim of the present study is to assess the status of trichomoniasis in China. To address this gap, we searched seven databases for relevant studies published from their inception to June 2024. The overall prevalence of *T. vaginalis* in China was determined to be 6.31% with a high level of heterogeneity (*I*^2^ = 99.68%). Subgroup analysis also demonstrated a statistically significant association between the *T. vaginalis* prevalence in the type of population, age range, year, residential status, and province. Among these, sex workers are the most prominent with 12.16%. Meta-regression analysis indicated that the infection rate of *T. vaginalis* among Chinese women had not shown a significant decline over time (*p* = 0.2919). Therefore, it continues to be a public health issue that should not be overlooked. Sex workers and rural women have a relatively higher infection rate of trichomoniasis, and this is largely associated with sexual safety awareness and hygiene conditions. Our findings provide crucial information for healthcare authorities and can shed light on the prevention strategies for trichomoniasis in China.

## 1. Introduction

*Trichomonas vaginalis* (TV) Donné, 1836 is a microaerobic flagellated parasitic protozoa that mainly infects the female vagina via sexual transmission [1]. Approximately half of the female infection cases are asymptomatic; however, about 50% of the infected women will present with symptoms such as increased vaginal discharge, itching, dyspareunia, frothy discharge, and dysuria [2]. In males, TV colonizes the urethra, epididymis, and prostate, but this infection is generally asymptomatic in males [3]. Trichomoniasis is a non-viral sexually transmitted infection (STI). According to data collected by the World Health Organization (WHO) in 2021, 156 million new cases are diagnosed annually [4]. In 2016, the global prevalence of trichomoniasis in women was 5.3% [5], which suggests that trichomoniasis is a high-risk STI.

*Trichomonas vaginalis* infection has also been linked to a range of more serious diseases, such as prostate cancer, adverse pregnancy outcomes, infertility, and cervical cancer [6]. Multiple studies showed that individuals infected with *T. vaginalis* have a greater risk of cervical cancer [7,8]. Based on a recent meta-analysis on cervical neoplasia, the researchers found a significant positive association between TV infection and the development of cervical neoplasia [8]. Moreover, the co-existence of TV and human immunodeficiency virus (HIV) can have serious consequences [9], and people with TV infection are 1.5 times more likely to be infected with HIV than people without TV infection [10]. Therefore, more efforts are needed to raise public awareness of TV infection and its potential health risks, as well as to promote preventive measures.

The degree of trichomoniasis infection varies in different countries and mainly depends on the local socioeconomic status, education level, and medical healthcare system. The prevalence of TV infection is geographically variable worldwide. Africa has the highest prevalence of infection (20.2% of females, 2% of males), and followed by the Americas (22% of females, 2.2% of males). Moving on to the Eastern Mediterranean region, the infection rate is 8% in females and 0.8% in males. In Europe, the infection rate is estimated to be 5.8% for females and 0.6% for males. Similarly to that in Southeast Asia, the infection rate there is 5.6% in women and 0.6% in men. In the Western Pacific region, the infection rates are almost the same, with 5.7% in females and 0.6% in males [4,11]. Currently, there are no studies on the overall infection rate in Asia. In China, published epidemiological investigations of TV infection are mainly focused on specific populations in certain areas, gynecological outpatient cases in hospitals, and unique populations, and no overall meta-analysis of women has been conducted. Therefore, we planned and carried out the present comprehensive systematic review and meta-analysis in order to offer the latest information about the prevalence of TV infection among Chinese women and to identify possible risk factors. To the best of our knowledge, this is the first study on the prevalence of trichomoniasis in China.

## 2. Materials and Methods

This systematic review and meta-analysis adhered to the Preferred Reporting Items for Systematic Reviews and Meta-Analyses (PRISMA) guidelines [12]. It was registered in PROSPERO with the registration number CRD42024538838.

### 2.1. Search Strategy

The results of our systematic review and meta-analysis are based on the PRISMA guidelines. To investigate the prevalence of *T. vaginalis* infection among women in the Chinese population, we performed a systematic search of the relevant literature released online in English and Chinese databases. Five English databases (PubMed, Science Direct, Web of Science, EBScohost, and Ovid MEDLINE medical literature library) and two Chinese databases (China National Knowledge Infrastructure (CNKI) and Wanfang Datebase) were used to search for articles about the prevalence of *T. vaginalis* among Chinese women from inception to 20 June 2024. The following key words were used to search the databases: *T. vaginalis*, Trichomoniasis, Epidemiology, Prevalence, Infection, China, and Chinese, either alone or in combination. The search strategy was listed in the Supplementary Text S1.

### 2.2. Selection Criteria

To screen for eligible studies, we developed the following criteria. (a) The target population was all adult women. (b) The purpose of the study was, or included, the infection rate of *T. vaginalis*. (c) The total number of participants exceeded 100. (d) The study included information on the number of examined populations and the number of positive individuals reported. Reviews and case reports were not included. Conference papers, books, graduate theses, and notes that did not meet our inclusion criteria were also excluded from this study.

### 2.3. Data Collection

The data were independently searched and extracted by two reviewers independently, and any disagreements were resolved via discussion with a third party. We utilized the duplicate—checking function of the literature management software Noteexpress to effectively eliminate duplicate entries, followed by manual verification. Through title-based screening, we removed articles irrelevant to the target population and diseases. Abstract-based screening further enabled us to discard articles related to gynecological diseases but not involving the target disease. For the studies that were included, we collected the first author, publication year, sampling year, sampling location, sample size, participant information, and number of TV positive individuals.

### 2.4. Literature Quality Assessment

To evaluate the quality of the studies selected, we used the standard Strengthening the Reporting of Observational Studies in Epidemiology (STROBE) checklist, which contains 22 sections [13]. This checklist includes all aspects of an article, such as objectives, study design, setting, participants, bias, study size, statistical methods, main results, other analysis, discussion, and funding information. Each item is scored with 0, 1, or 2 points: 0 indicates that the relevant content is not mentioned; 1 point indicates that it only mentions relevant content without elaborating; and 2 points indicate that the text explains the relevant content in detail. The full score is 44 points. References with a score <17.5 were considered low quality; references with a score of 17.5–35 were classified as medium quality; and references with a score of 35–44 were classified as high quality.

### 2.5. Statistical Analyses

The Wilson method was used to estimate the prevalence of *T. vaginalis* and its 95% confidence interval (CI) for each study. The *I*^2^ statistic and Cochran’s Q were used to evaluate the heterogeneity among studies (*p* < 0.1 indicated statistical significance). Based on the Higgins classification method, an *I*^2^ value higher than 70% was determined as high heterogeneity [14]. We used a random effects model to estimate the pooled values with high heterogeneity. Furthermore, forest plot analysis was provided for subgroup analyses stratified by population type, age, sample year, publication year, urbanization status, and geographic area, to investigate between study sources of heterogeneity. We also performed a leave-one-out sensitivity analysis to further examine the possible causes of heterogeneity among the studies involved in the analysis. All the meta-analyses were performed using STATA statistical software 17 developed by StataCorp LLC (2023) and Meta-XL.

## 3. Results

### 3.1. Study Inclusion Criteria

**Figure 1:**
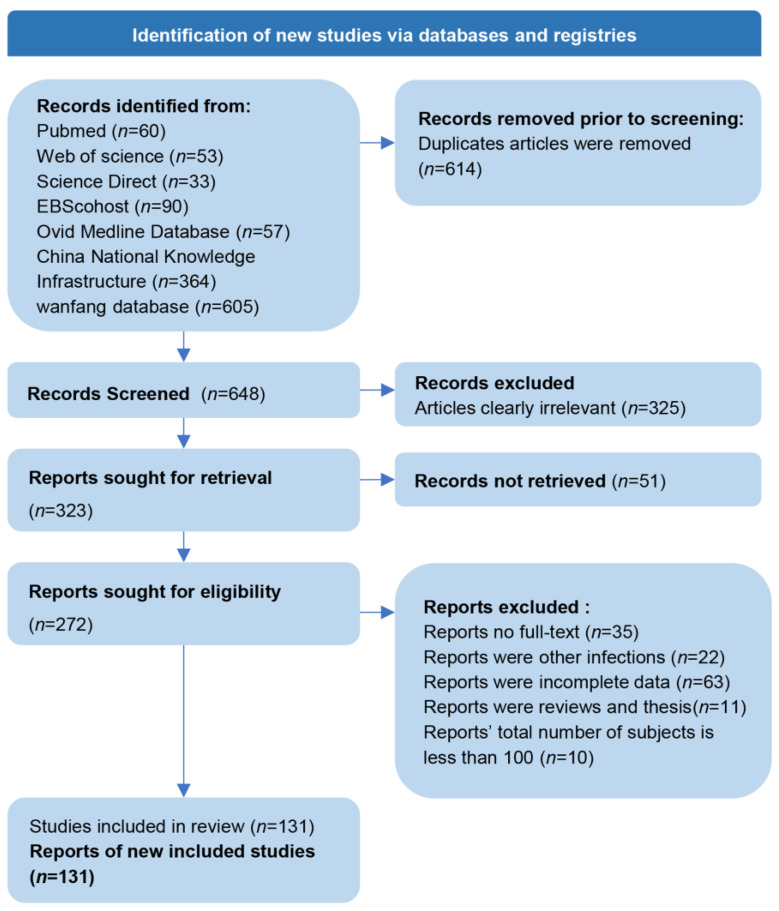
Flow diagram for the screening of the included studies.

By searching the literature database, we obtained a total of 1262 publications (293 in English, 969 in Chinese), 272 of which remained after removing the duplicate studies and a preliminary screening. Thirty-five of these articles did not have full text; 22 articles covered vaginal infections in animals; 63 articles had incomplete data; 11 articles were retrospective; and the total number of subjects in 10 articles was less than 100. After full-text review, 131 studies focusing on *T. vaginalis* were ultimately included based on the stringent inclusion criteria (Figure 1) [15,16,17,18,19,20,21,22,23,24,25,26,27,28,29,30,31,32,33,34,35,36,37,38,39,40,41,42,43,44,45,46,47,48,49,50,51,52,53,54,55,56,57,58,59,60,61,62,63,64,65,66,67,68,69,70,71,72,73,74,75,76,77,78,79,80,81,82,83,84,85,86,87,88,89,90,91,92,93,94,95,96,97,98,99,100,101,102,103,104,105,106,107,108,109,110,111,112,113,114,115,116,117,118,119,120,121,122,123,124,125,126,127,128,129,130,131,132,133,134,135,136,137,138,139,140,141,142,143,144,145]. Basic information about the included studies is presented in Supplementary Table S1, and they were classified into four categories according to the subject population types: general women (37 articles), hospital gynecological outpatient patients (84 articles), pregnant women (4 articles), sex workers and sex offenders (6 articles). The quality assessment revealed that 21 (15.67%) articles were of high quality (≥35), 103 (76.87%) articles were of moderate quality (≥17.5~35), and 10 (7.46%) articles were of low quality (<17.5).

### 3.2. Meta Analysis of the Epidemiological Investigations

**Figure 2:**
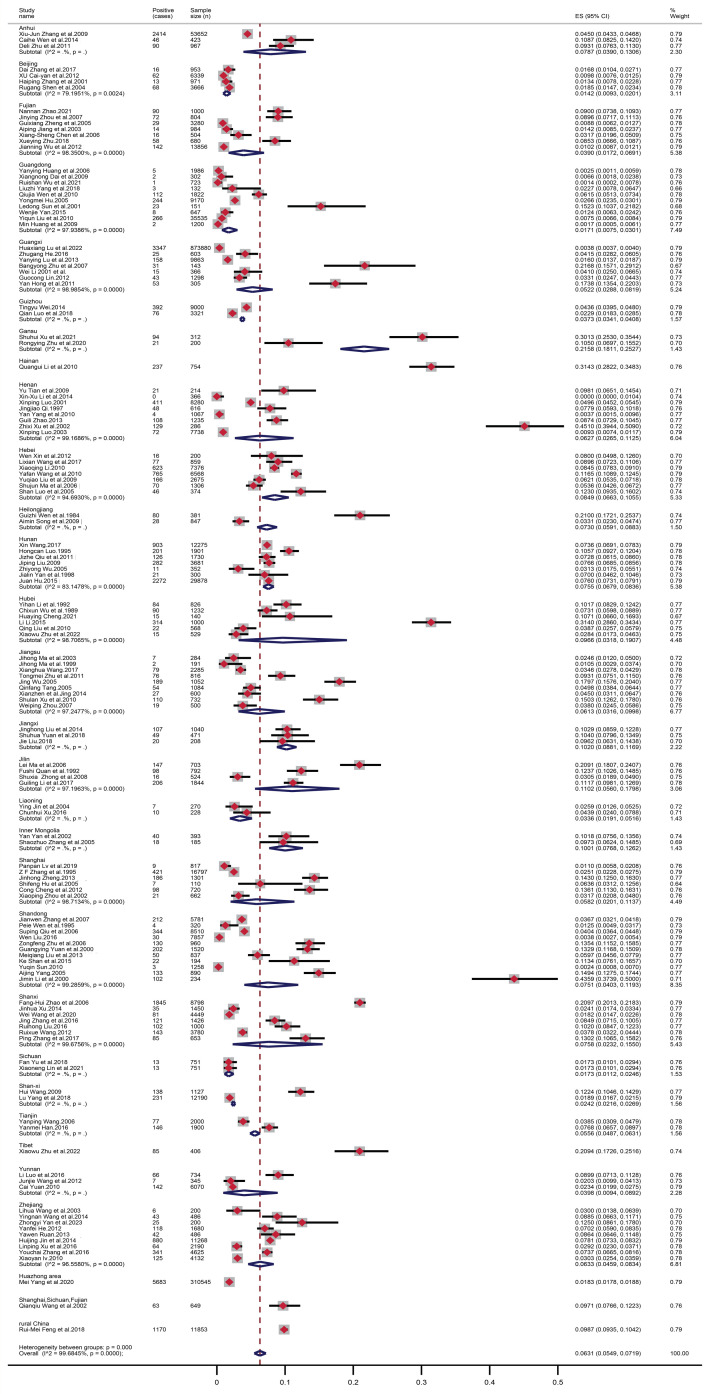
Forest plots for the random-effects meta-analysis of *T. vaginalis* infection among women in the Chinese population. The boxes indicate the effect size of the studies (prevalence), with whiskers indicating the corresponding confidence intervals (Cis). For black hollow diamonds, the size reflects the magnitude of the effect, and the length of the diamonds represents the CIs [15,16,17,18,19,20,21,22,23,24,25,26,27,28,29,30,31,32,33,34,35,36,37,38,39,40,41,42,43,44,45,46,47,48,49,50,51,52,53,54,55,56,57,58,59,60,61,62,63,64,65,66,67,68,69,70,71,72,73,74,75,76,77,78,79,80,81,82,83,84,85,86,87,88,89,90,91,92,93,94,95,96,97,98,99,100,101,102,103,104,105,106,107,108,109,110,111,112,113,114,115,116,117,118,119,120,121,122,123,124,125,126,127,128,129,130,131,132,133,134,135,136,137,138,139,140,141,142,143,144,145].

**Figure 3:**
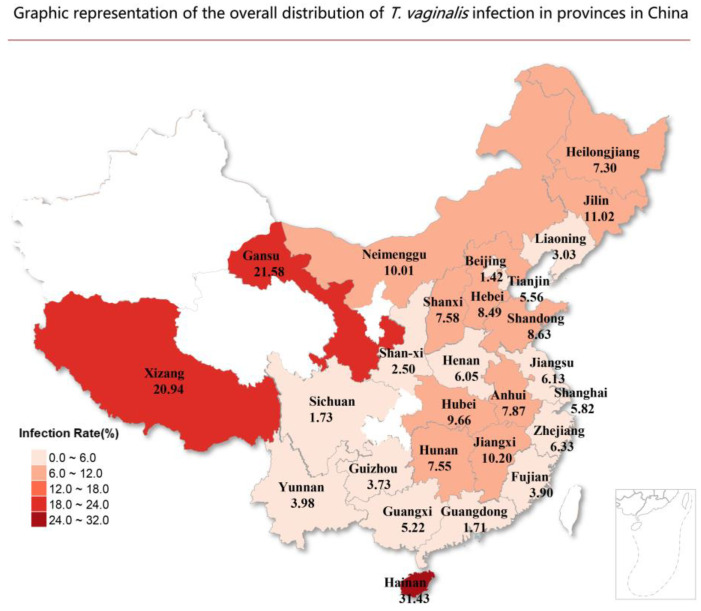
Graphic representation of the overall distribution of *T. vaginalis* infection in provinces in China. The *T. vaginalis* infection rate is shown in different colors.

From 1984 to 20 June 2024, a total of 1,593,706 Chinese women were examined regarding TV infection, resulting in a calculated combined prevalence of 6.31%, which was estimated with random effects (*I*^2^ = 99.68%) (Figure 2). We used the Standard Map Service (http://bzdt.ch.mnr.gov.cn/, (accessed on 18 April 2025)) to generate a map that depicts the prevalence of TV in each province of China, based on the included studies (Figure 3). Among the provinces, most examinations were conducted among women in Shandong and Guangdong, with 11 (7.51%) and 10 (1.71%) studies, respectively. The highest prevalence rate was in Henan (45.10%) [134]. Second, patients from the hospital’s gynecological department had a high prevalence rate in Shandong [144], Hainan (*n* = 1) [86], Hubei (*n* = 3) [74] and Gansu (*n* = 2) [55], with 43.59%, 31.43%, 31.40% and 30.13%, respectively. The lowest prevalences were reported from Beijing (1.42%), Guangdong (1.71%) and Sichuan (1.73%).

Subgroup analysis was further conducted to figure out the weighted prevalence and minimize heterogeneity with respect to 11 different parameters, which include age, year, development level, residential status, city, prisoners or sex workers, patients from the gynecological clinic, pregnancy status, and ordinary women (Table 1). As expected, groups of prisoners or sex workers and patients from the gynecological clinic showed a significantly higher prevalence rate of TV than the general population of women and pregnant women, with 12.16%, 8.01%, 3.48%, and 2.54%, respectively, meaning that the prevalence of symptomatic infection was higher than asymptomatic infections. The general population of women can be considered as asymptomatic carriers to a certain extent, as they are found to be infected with TV only during mass screening or physical examinations. This indicates that the infection rate of pathogenic TV is significantly higher than that of non-pathogenic TV. The prevalence rate was higher in rural areas (6.80%) than in urban settings (4.60%); there is no significant difference between north (7.84%) and south (7.67%) China. Regardless of publication or sample collection years, the infection rate of TV in Chinese women before 2000 was significantly higher than that after 2000. However, since 2020, TV infection rate has increased again. This may be because there was only one study (by sample year) at that stage [118], and this study was not representative. In addition, we assessed the rate of trichomoniasis infection among women of different ages according to 46 studies which had detailed age-group infection rates. The prevalence of trichomoniasis was 0.41% in women under the age of 20 years, 6.93% in women aged 20~30 years, 10.46% in women aged 31~40 years, 9.42% in women aged 41~50 years, 5.47% in women aged 51~60 years, and 4.94% among those older than 60 years. The data showed that the percentage of *T. vaginalis*-positive individuals was significantly greater in women aged 30–50 years than in other age groups.

### 3.3. Publication Bias, Sensitivity Analysis, and Meta-Regression

**Figure 4:**
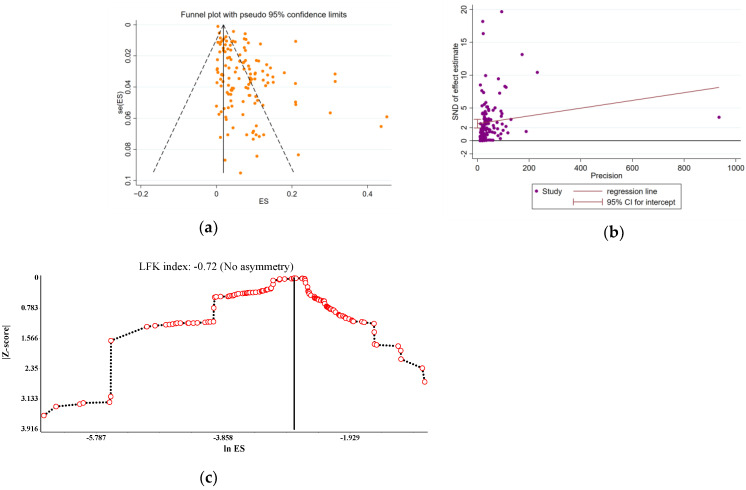
(**a**) Funnel plot and (**b**) Egger’s test plot were utilized to evaluate the publication bias within the current study. The colored circles stand for each individual study, the middle line indicates the effect size, while the other two lines represent the corresponding confidence ranges. (**c**) Doi plot for the prevalence of TV infection among the female population of China. A Luis Furuya-Kanamori (LFK) index of 0.72 indicates major asymmetry.

**Figure 5:**
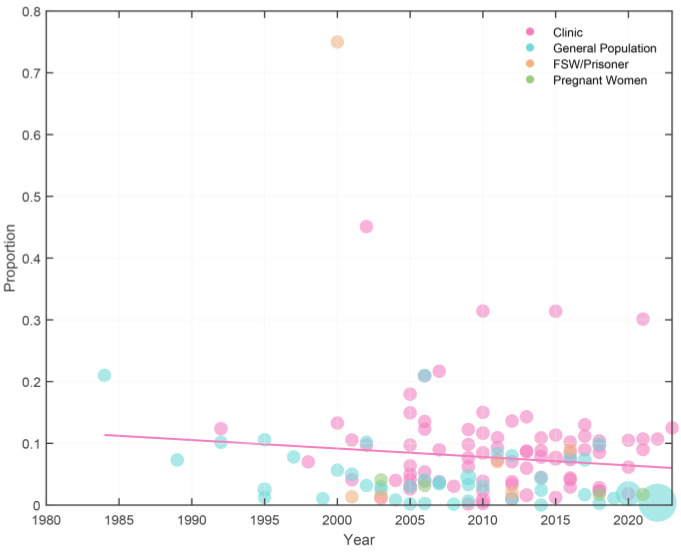
A meta-regression graph regarding the prevalence of trichomoniasis among Chinese female population in accordance with the year of publication: the pink line is the regression line, which was plotted based on the intercept and the slope of the regression model. The bubbles of different colors symbolize the type of population, and their sizes denote the effect size of each study.

We performed a leave-one-out sensitivity analysis to further examine the possible causes of heterogeneity among the studies involved in the analysis. This analysis demonstrated that the results of the primary analysis are robust and do not rely on any single study (Supplementary Figure S1). A highly significant publication bias was detected using the Funnel plot and Egger’s regression plot. However, a more robust alternative method based on specific statistical principles, the Doi plot, was employed. The Doi plot showed no asymmetry (LFK index: 0.72), meaning that no publication bias exists (Figure 4). Moreover, our meta-regression analysis failed to identify a statistically significant correlation between the prevalence and the year of publication (*p* = 0.2919) (Figure 5) [146].

## 4. Discussion

*Trichomonas vaginalis* is a highly prevalent STI with nearly 400 million cases worldwide [147]. Epidemiological studies contribute to the design and evaluation of preventive care strategies and patient services. However, the results of sporadic epidemiological studies may be controversial and inconsistent, which poses inconvenience to the clinical management of disease. Therefore, systematic reviews can help to overcome this problem. Previously in China, Yang et al. [7] and Mei et al. [148] performed a meta-analysis of TV infection-associated risk of cervical cancer in 2018 and in 2023, respectively. Zhang et al. published a systematic review of the correlation between TV infection and infertility in 2022 [149], and reproductive system cancer in 2023 [150]. To our knowledge, this is the first meta-analysis of trichomoniasis prevalence in Chinese women, and this study may provide information that will inform actions to improve public health.

The studies included in this paper were published from 1984 to 2024, with a total sample size of 1,593,706 women in 27 provinces, of which 30,453 individuals were positive for trichomoniasis. We used the metaprop function to construct the forest map because there were extreme values in the included studies (the infection rate was 0) [46]. The pooled prevalence among women in the China was 6.31%, which is significantly greater than that in developed countries, such as the United States (1.8%) [151], England (1.27%) [152], and France (1.7%) [153]. This finding might be attributed to differences in geographical regions, income levels, age, type of population, or habits reflected in these studies. Therefore, TV is still a severely neglected disease, especially in some provinces and autonomous regions where relevant investigations were not performed. The results of the *I*^2^ test revealed high heterogeneity in the prevalence of trichomoniasis in different provinces of China among the eligible studies. The results of subgroup analysis indicated that population type, age, and province might be the causes of heterogeneity. Infection rate among pregnant women was relatively low, probably due to reduced sexual activity and greater attention to personal hygiene during pregnancy. On the other hand, it is also plausible that the limited number of published articles (*n* = 4) may not accurately reflect the true prevalence of TV infection among pregnant women in China. There is a limitation that we considered positive women found during mass screening or physical examinations as asymptomatic carriers. For the general female population, there have been few large-scale screenings at the provincial level or above in China. In contrast, health surveys and free medical examinations carried out at the county and town levels, along with those in community settings, are more prevalent. We speculate that the positive patients identified through screening are asymptomatic. In contrast, infected individuals with clinical symptoms would seek medical examination and treatment at hospitals and thus fall into the category of cases admitted to hospital outpatient departments. This is a rough speculation. There may be some women who, despite experiencing symptoms, are still able to tolerate them and do not seek treatment.

The low infection rate among women under 20 years old might be related to the Chinese national conditions, such as limited education on sexual relationships and late awakening of sexual awareness. The economic development levels and the distribution of healthcare resources vary among different provinces. Provinces with developed economies usually have more complete healthcare systems and residents with higher health awareness, resulting in a potentially lower infection rate. In contrast, in provinces with relatively backward economies, limited healthcare resources, and insufficient popularization of health knowledge among residents may lead to a higher infection rate. After subdividing into subgroups, the infection situations within the same group were relatively more consistent, which decreased heterogeneity.

Sex workers, due to their frequent sexual contacts and high-risk behaviors, are a high-risk group for STIs. The pooled prevalence of TV among female sex workers globally is 16% [154], and the positive rate in the Veracruz region of central Mexico is 25.7% [155]. Our findings indicated that the infection rate of sex workers and sexual offenders is significantly higher (12.16%) than that of other specific populations. Especially a study carried out in 2000 in Shandong, which reported that the infection rate of TV was as high as 75% [145]. However, this infection rate among sex workers and sexual offenders in our study is lower than the global average infection rate. Sex workers represent a high-risk population for STIs. Frequent sexual encounters not only increase the likelihood of transmitting infections to clients but also amplify the risk of spread due to the multiple sexual partners sex workers typically have. Studies indicate that approximately half of TV-infected women are asymptomatic. Moreover, TV infection increases the risk of acquiring other infections, such as HIV and *human papillomavirus* (HPV), as well as adverse pregnancy outcomes, thereby posing a significant threat to public health. In addition, the high prevalence of trichomoniasis in gynecological clinics suggests that TV still occupies a certain position in sexually transmitted diseases. Our review covered 24 provinces and three municipalities. However, there is only one study in Hainan (31.43%) and Tibet (20.94%), respectively, and these rates may not be representative. In total, thirteen provinces and municipalities have more than three studies, with Shandong (7.51%, *n* = 11) and Guangdong (1.71%, *n* = 10) having the most. Six provinces had an infection rate of TV greater than 10%, including Hainan (31.43%, *n* = 1), Gansu (21.58%, *n* = 2), Tibet (20.94%, *n* = 1), Jilin (11.39%, *n* = 4), Jiangxi (10.20%, *n* = 3), Inner Mongolia (10.01%, *n* = 2). These six provinces have lower study numbers. Therefore, this might not represent the true prevalence of TV infection within these provinces. The pooled TV infection rate in eleven provinces was greater than 5% and less than 10%, accounting for 40.74% of the total.

We screened studies that mentioned the residency status of the subjects, and the analysis showed that urban cities with high income levels had a lower prevalence of *T. vaginalis* (4.60%) compared to rural counties (6.80%). One potential explanation for this pattern is that the urban population has a cleaner living environment and better public health promotion. Regardless of the years of publication or sample collection, the infection rate of TV in Chinese women before 2000 (10.66%) was significantly higher than that after 2000, which may be due to improvements in living standards, medical conditions, education levels and self-hygiene awareness. The pooled infection rate from the year 2000 to 2010 (5.80%), and there was no significant change compared with that from 2010 to 2020 (5.76%). However, the infection rate has risen since 2020 (7.20%), because, according to the sample time, there was only one study during this period, and it is not representative and therefore can be ignored. With widespread economic and social changes, people’s lifestyles have changed dramatically, and their behaviors and minds have become more open. Also, the diversity of entertainment venues has facilitated the spread of sexually transmitted diseases. For example, heterosexual transmission of HIV has become the main mode of HIV transmission in China, accounting for 62.6% of acquired immunodeficiency syndrome (AIDS) cases in 2009, compared to 11.3% before 2006. Therefore, trichomoniasis, which is a sexually transmitted disease, should not be ignored [156].

The aggregated rates of TV infection in different age groups were derived from studies with detailed subgroups (*n* = 46). The analysis indicated that the prevalence of *T. vaginalis* was higher in women aged 31 to 40 (10.46%, *n* = 41) and 41 to 50 (9.42%, *n* = 41) than in women aged 21–30 years (6.93%, *n* = 38), ≤20 years (0.41%, *n* = 14), 51 to 60 years (5.47%, *n* = 34) or ≥60 years (4.94%, *n* = 12). People aged 30 to 50 are classified as young and middle-aged, and these populations are energetic and have rich lives. Moreover, individuals in these people are also sexually active and sexual transmission is the most common transmission route for infection. Men generally have no obvious symptoms after infection and are more likely to become a source of transmission to women due to unprotected sexual activities. Trichomoniasis is often diagnosed in older women. In our study, the infection rate among the elderly was relatively low, which was consistent with the previous report by Obetta KC et al. [157]. This could be ascribed to the reduced likelihood of sexual transmission resulting from their relatively low sexual activity. However, a study carried out in the United States revealed that, in comparison to younger cohorts, elderly female patients exhibited a higher susceptibility to trichomoniasis [158]. Such a divergence may be attributable to disparities in multiple aspects among different countries, including healthcare availability, sexual health-related educational policies, and sociocultural norms. Moreover, the decline in estrogen levels in the elderly population renders the vaginal microbiota more prone to imbalance, thereby creating opportunities for the infection of *T. vaginalis*.

Trichomoniasis is a health problem in most countries. For instance, in 2016, there were 156 million new cases, which is equivalent to 40 infections per 1000 women [159]. The results of a study on women in the general Iranian population revealed an 8% incidence of trichomoniasis infection [160]. In contrast, from 2002 to 2020, the incidence of trichomoniasis infection in Turkey was 6.17% [161]. In 1999, South Korea reported a 10.4% infection rate [162]. Japan had an incidence of 4.2% [163]. In 2024, the Tabriz region of Iran reached 6.8% [164]. Our findings are consistent with the average prevalence of infection worldwide (5–10%). The high infection rate observed in China is closely associated with the country’s socioeconomic status, traditional sexual values, and inadequate sex education. Our results demonstrated a pronounced publication bias. This was mainly because the infection rate presented a positive outcome without corresponding negative results for counterpoise, consequently giving rise to the asymmetry within the funnel plot. The meta-regression analysis did not find a statistically significant correlation between prevalence and year of publication. On the one hand, this may be attributed to the restricted number of included studies and the inherent limitations of the samples. On the other hand, although people’s health awareness has been continuously enhanced, other confounding factors might have interfered, resulting in the failure to show the expected change in prevalence over time in the meta-regression analysis. For the treatment of trichomoniasis, metronidazole (MTZ) and Tinidazole (TNZ) have always been the classic medications [165]. Although MTZ-resistant strains have been reported since 1962, the incidence of drug-resistant cases remained low until the late 1990s, when a sharp increase in the number of MTZ treatment failures was noted [166]. This is one of the reasons why infection rates have not declined, demonstrating once again that trichomoniasis remains a public health problem to which we should pay attention. Fortunately, secnidazole (SEC), which has been newly approved by the Food and Drug Administration (FDA) for trichomoniasis treatment, demonstrates superior pharmacokinetic properties. This offers additional prospects for achieving a cure for trichomoniasis [167].

One limitation of the present study is that many provinces and cities never surveyed the prevalence of *T. vaginalis* infection among women in the general population, or there were only one or two studies for one region, which is not representative of the prevalence of the entire province. The lack of sex education makes people naturally reject related topics, and some asymptomatic infected people refuse to be examined. Notably, it is estimated that approximately 50% of women infected with TV are asymptomatic [168]. Although there are very few epidemiological investigations of TV infection in men in China, some studies from other countries reported interesting results. 77.3% of TV infections in men are asymptomatic, and this asymptomatic state can last for months. Men are important vectors of transmission to women [159]. This contributes to neglect of the diagnosis and treatment of TV and makes subsequent developments more difficult to track. Trichomoniasis is currently not a reportable infection in developed or developing countries. This situation has hindered the collection of meaningful epidemiological data on TV infection [169]. Therefore, the estimation of TV incidence seems incomplete, yet it reveals relative infection rates in the country. More investigations are needed to accurately determine the prevalence across China and the infection status of urban and rural high-risk groups of men and women.

The results of studies presented in China were mostly based on microcopy diagnostic techniques, but the sensitivity ranged from 60 to 80%, and there is still the possibility of missing detection [170]. Of all the included studies, only one paper used the culture method, although the accuracy of the culture method is 100%, it is too long and rarely used in the clinic. Molecular-based techniques, such as PCR, are more sensitive and specific, resulting in lower rates of misdiagnosis and missed diagnosis. Nevertheless, due to their complicated operation steps and high cost, they have not been widely adopted in the clinic. Therefore, the identification of trichomoniasis in China may underestimate its true prevalence.

## 5. Conclusions

In conclusion, our comprehensive and systematic analysis regarding *T. vaginalis* infection among Chinese women showed a significantly higher infection rate compared with that in developed countries. Despite the relatively more conservative sexual attitudes among the Chinese population compared with those in European and American countries, the stigma surrounding sex education and related discussions has, to a certain extent, impaired people’s awareness of sexually-transmitted diseases, thereby exacerbating the spread of such diseases. It continues to be a significant health issue among the Chinese population, particularly in rural areas and special populations. Prevalence depends on the socio-economic structure of the area, the lifestyles of individuals, and the age and size of the population. The decrease in the TV infection rate after 2000 was most related to improvements in people’s health awareness. The data provide support for the mass screening of individuals and show a high incidence of TV infection in women aged 30 to 50 years. The results also support the need for increased awareness among young women about modes of TV transmission. To control infection, we need to conduct hygiene education, select appropriate diagnostic methods, and treat positive patients. Overall, this review provides valuable information on the epidemiology of TV in the Chinese population and may be helpful for infection management and control procedures.

## Figures and Tables

**Table 1:** Basic characteristics of eligible studies reporting the prevalence of trichomoniasis from China.

Variables	Number of Studies	Sample Size	Positive Cases	Pooled Prevalence (95% CI)	*I* ^2^	*τ* ^2^	*p*
Population type							
general	38	1,358,641	18,390	0.0348 (0.0260, 0.0448)	99.81%	0.0299	*p* < 0.001
clinic	83	225,820	11,698	0.0801 (0.0678, 0.0933)	99.11%	0.0464	*p* < 0.001
pregnant	4	5672	110	0.0254 (0.0155, 0.0377)	68.67%	0.0034	*p* = 0.0225
FSW and prisoner	6	3573	255	0.1216 (0.0318, 0.2570)	99.02%	0.1338	*p* < 0.001
Residential status							
Urban	13	365,417	3048	0.0460 (0.0266, 0.0703)	99.53%	0.0348	*p* < 0.001
Rural	13	639,629	8271	0.0680 (0.0319, 0.1161)	99.92%	0.0925	*p* < 0.001
Publish year							
≤2000	13	25,110	1353	0.1004 (0.0596, 0.1502)	98.80%	0.08	*p* < 0.001
2001~2010	53	201,457	10,015	0.0850 (0.0652, 0.1071)	99.65%	0.1	*p* < 0.001
2011~2020	57	486,802	15,368	0.0583 (0.0480, 0.0694)	99.30%	0.0319	*p* < 0.001
2021~2024.05	8	877,941	3685	0.0720 (0.0235, 0.1435)	99.21%	0.1235	*p* < 0.001
Sample year							
≤2000	20	36,282	2032	0.0933 (0.0662, 0.1244)	98.53%	0.055	*p* < 0.001
2001~2010	66	232,360	11,581	0.0910 (0.0718, 0.1123)	99.64%	0.0903	*p* < 0.001
2011~2020	52	1,322,368	16,780	0.0539 (0.0438, 0.0649)	99.72%	0.0289	*p* < 0.001
2021~2024.05	1	200	25	0.1250 (0.0861, 0.1780)	— ^1^	— ^1^	— ^1^
Area							
Southern	77	1,150,874	16,944	0.0767 (0.0627, 0.0919)	99.72%	0.0686	*p* < 0.001
Northern	52	122,086	8040	0.0784 (0.0596, 0.0994)	99.39%	0.0793	*p* < 0.001
Age							
≤20	14	1040	39	0.0041 (0.0000, 0.0270)	76.42%	0.0700	*p* < 0.001
20~30	38	28,061	2151	0.0693 (0.0441, 0.0996)	98.75%	0.1400	*p* < 0.001
31~40	41	288,662	5255	0.1046 (0.0690, 0.1464)	99.71%	0.2000	*p* < 0.001
41~50	41	360,652	5378	0.0942 (0.0606, 0.1340)	99.66%	0.1600	*p* < 0.001
51~60	34	201,453	2391	0.0547 (0.0210, 0.1012)	99.47%	0.2200	*p* < 0.001
≥60	12	47,597	583	0.0494 (0.0003, 0.1484)	99.45%	0.3000	*p* < 0.001
Province							
Anhui	3	55,042	2550	0.0787 (0.0390, 0.1306)	— ^1^	0.0192	*p* < 0.001
Beijing	4	11,929	159	0.0142 (0.0093, 0.0201)	79.20%	0.0016	*p* < 0.001
Fujian	7	24,694	426	0.0390 (0.0172, 0.0691)	98.40%	0.0324	*p* < 0.001
Guangdong	10	51,754	668	0.0171 (0.0075, 0.0301)	97.93%	0.0166	*p* < 0.001
Guangxi	7	886,564	3648	0.0522 (0.0288, 0.0819)	98.99%	0.0196	*p* < 0.001
Guizhou	2	12,321	468	0.0373 (0.0341, 0.0408)	— ^1^	— ^1^	*p* < 0.001
Gansu	2	512	115	0.2158 (0.1811, 0.2527)	— ^1^	— ^1^	*p* < 0.001
Hainan	1	754	237	0.3143 (0.2822, 0.3483)	— ^1^	— ^1^	*p* < 0.001
Henan	8	28,040	889	0.0627 (0.0265, 0.1125)	98.63%	0.0559	*p* < 0.001
Hebei	7	19,358	1763	0.0849 (0.0663, 0.1055)	94.69%	0.0077	*p* < 0.001
Heilongjiang	2	1228	108	0.0730 (0.0591, 0.0883)	— ^1^	— ^1^	*p* < 0.001
Hunan	7	50,117	3816	0.0755 (0.0679, 0.0836)	83.15%	0.0452	*p* < 0.001
Hubei	6	4295	540	0.0966 (0.0318, 0.1907)	98.71%	0.1120	*p* < 0.001
Jiangsu	9	7544	563	0.0613 (0.0316, 0.0998)	97.25%	0.0449	*p* < 0.001
Jiangxi	3	1719	176	0.1020 (0.0881, 0.1169)	0.00%	0.0000	*p* = 0
Jilin	4	3758	467	0.1102 (0.0560, 0.1798)	97.20%	0.0318	*p* < 0.001
Liaoning	2	498	17	0.0336 (0.0191, 0.0516)	— ^1^	0.0059	*p* < 0.001
Inner Mongolia	2	578	58	0.1001 (0.0768, 0.1262)	— ^1^	— ^1^	*p* < 0.001
Shanghai	6	20,407	742	0.0582 (0.0201, 0.1137)	98.71%	0.0597	*p* < 0.001
Shandong	11	28,361	1232	0.0751 (0.0403, 0.1193)	99.27%	0.0625	*p* < 0.001
Shanxi	7	21,556	2412	0.0758 (0.0232, 0.1550)	99.68%	0.1143	*p* < 0.001
Sichuan	2	1502	26	0.0173 (0.0112, 0.0246)	— ^1^	— ^1^	*p* < 0.001
Shan-xi	2	13,317	379	0.0242 (0.0216, 0.0269)	— ^1^	— ^1^	*p* < 0.001
Tianjin	2	3900	223	0.0556 (0.0487, 0.0631)	— ^1^	— ^1^	*p* < 0.001
Tibet	1	406	85	0.2094 (0.1726, 0.2516)	— ^1^	— ^1^	*p* < 0.001
Yunnan	3	7149	215	0.0398 (0.0094, 0.0892)	— ^1^	0.0310	*p* = 0.0004
Zhejiang	9	25,267	1644	0.0633 (0.0459, 0.0834)	96.56%	0.0122	*p* < 0.001

^1^ “—” no data.

## Data Availability

The data supporting the conclusions of this article are included within the article.

## Supplementary Materials

The following supporting information can be downloaded at: https://www.mdpi.com/article/10.3390/tropicalmed10040113/s1, Text S1: Search strategies in different databases; Table S1: Basic characteristics of eligible studies reporting the prevalence of trichomoniasis from China; Figure S1: Leave-one-out sensitivity analysis demonstrated that the results of the primary analysis are robust and do not rely on any single study.

## Abbreviations

The following abbreviations are used in this manuscript:

*T. vaginalis* or TV	Trichomonas vaginalis
STI	sexually transmitted infection
PRISMA	Preferred Reporting Items for Systematic Reviews and Meta-Analyses
STROBE	Strengthening the Reporting of Observational Studies in Epidemiology
HIV	human immunodeficiency virus
MTZ	metronidazole
TNZ	Tinidazole
FDA	Food and Drug Administration
CI	confidence interval
CNKI	China National Knowledge Infrastructure
*d.f.*	distribution function
FSW	female sex worker
*I*^2^	heterogeneity
*τ*^2^	tau-squared
