# Supplementary figures and images for: Prevalence of Trichomonas vaginalis Among Women in the Chinese Population: A Systematic Review and Meta-Analysis
Source: Trop Med Infect Dis. 2025 Apr 19;10(4):113. doi: 10.3390/tropicalmed10040113 (PMC12031445; doi:10.3390/tropicalmed10040113)

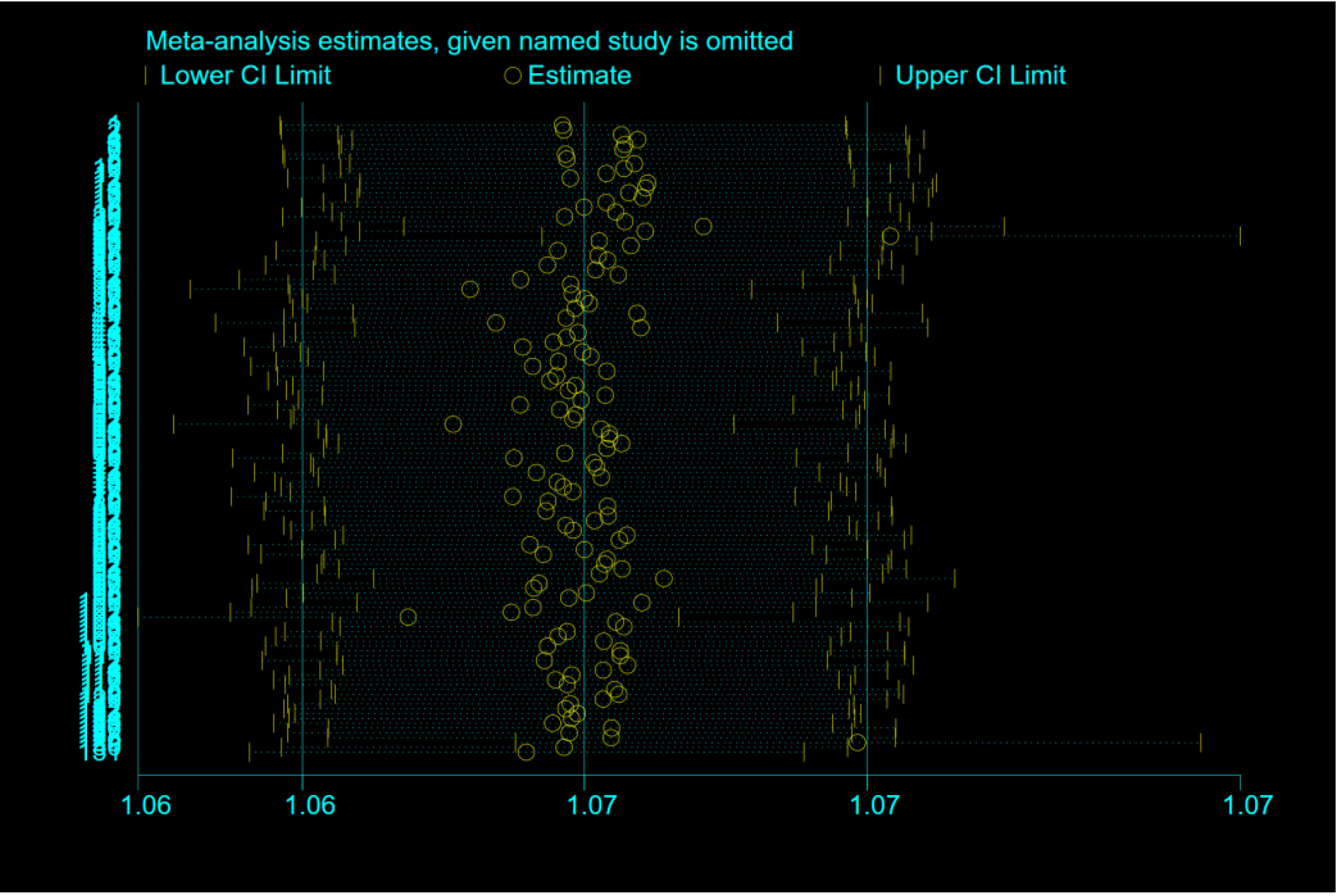

Supplement: Supplementary file 1 [file tropicalmed-10-00113-s001.zip › Supplementary/Supplementary Figure S1.tif]
